# Reactive perforating collagenosis and systemic lupus erythematosus: A rare case report

**DOI:** 10.1097/MD.0000000000032138

**Published:** 2022-12-02

**Authors:** Fahidah Alenzi

**Affiliations:** a Clinical Sciences Department, College of Medicine, Princess Nourah bint Abdulrahman University, Riyadh, Saudi Arabia.

**Keywords:** Kyrle disease, reactive perforating collagenosis, systemic lupus erythematosus, undifferentiated connective tissue disease, vasculitis

## Abstract

Transepidermal clearance of altered collagen and excessive excretion of keratin are characteristics of a rare cutaneous disorder known as reactive perforating collagenosis (RPC). There are different forms of RPC; however, the acquired form is the most prevalent and inherited. Reactive perforating collagenosis is rarely described in autoimmune rheumatic diseases; instead, it is typically linked to systemic conditions such as renal failure or hepatic disease.

**Methods:**

A 31-year-old Saudi female patient who was initially diagnosed with undifferentiated connective tissue disease. She developed RPC with a severe diffuse itchy skin rash with numerous papules and nodules with central hyperkeratotic plugs over the lower limb, upper limb, and face.

**Results:**

The patient tested positive for antinuclear antibody; however, a year later, patient developed Raynaud’s phenomenon, oral and nasal ulcers, malar rash, fatigue, and lupus rash around her eyes, and systemic lupus erythematosus was diagnosed clinically. The patient was treated for reactive perforating collagenosis with systemic antihistamines (diphenhydramine 50 mg orally twice daily), topical steroid cream (betamethasone dipropionate cream), and oral isotretinoin (20 mg daily). The patient was advised to undergo phototherapy. A year later, she presented with symptoms of systemic lupus erythematosus and started taking oral hydroxychloroquine 200 mg twice daily for systemic lupus erythematosus. The patient is listed on follow-up.

**Conclusion:**

Variable skin rash can mimic systemic lupus erythematosus and vasculitis. Therefore, reactive perforating collagenosis is a skin condition that requires high clinical suspension for diagnosis, and it might be challenging to determine whether it is an association or a complication. Furthermore, the timing of the skin biopsy may be crucial for the diagnosis of reactive perforating collagenosis.

## 1. Introduction

Reactive perforating collagenosis (RPC) is a rare cutaneous condition characterized by the trans-epidermal elimination of altered collagen and excessive excretion of keratin. There are different forms of RPC: acquired and inherited. Kyrle’s disease and elastosis are rare types of perforating collagenosis.^[1]^ The primary symptom of the disease, which is typically painless but frequently results in extremely itchy lesions, is the development of many papules and nodules with hyperkeratotic plugs that affect every part of the body, particularly the lower limbs.^[2,3]^ RPC primarily affects adults; however, it can occur in pediatric patients. Although individuals aged between 30 and 75 years are more susceptible to this condition, a case in a patient aged 5 years has been reported and recorded.^[4]^ Additionally, RPC is highly prevalent among women, with fewer cases being reported in men.^[5]^ Most lesions regress spontaneously within 6 to 8 weeks with post-inflammatory changes such as hypopigmentation or hyperpigmentation. However, most patients experience relapsing and remitting lesions throughout their lives. RPC is rarely associated with connective tissue diseases; however, it has been associated with a number of conditions including hyperlipoproteinemia, congestive heart failure, diabetes mellitus, hepatitis, and renal failure.^[1–4]^ A comprehensive understanding of the actual cause remains a considerable challenge. Herein, we describe a patient who was initially diagnosed with undifferentiated connective tissue disease and developed severe diffuse itchy skin rash with numerous papules and nodules with a central keratotic plug over her lower limb, upper limb, and face with a positive anti-nuclear antibody. However, a year later, she presented with symptoms of systemic lupus erythematosus (SLE).

## 2. Consent for publication

The patient provided a written informed consent to publish the case.

## 3. Case report

**Figure 1.**
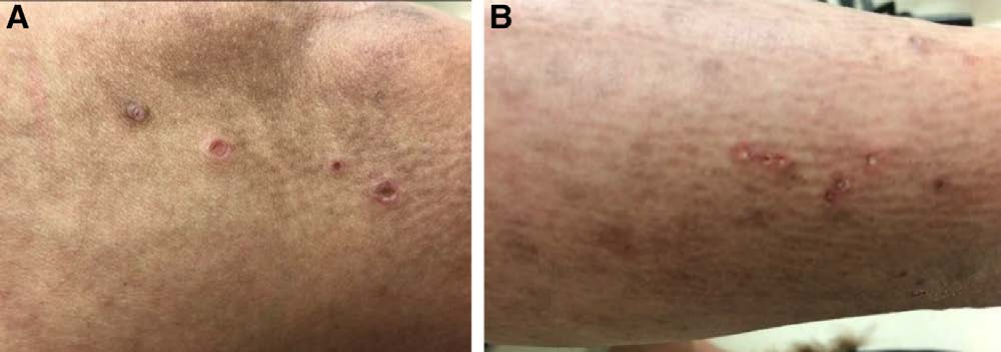
(A) and (B) showed scattered and numerous papules and nodules with central keratotic plug over her lower limb, upper limb and back.

**Figure 2.**
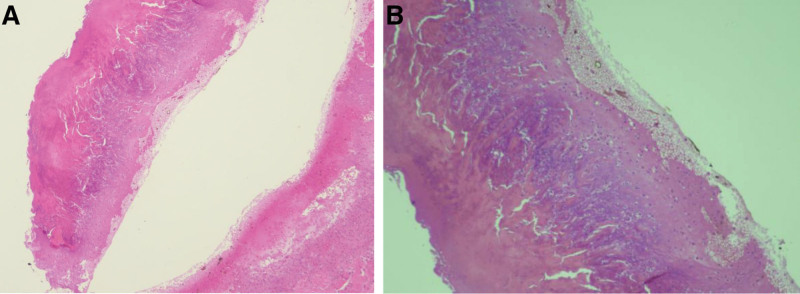
(A) and (B) skin tissue biopsy showed fibrin material in papillary dermis and serum crust, Trichrome and Verhoeff’s stain stains show collagen and elastin fibers in keratin layer. No evidence of vasculitis.

A 31-year-old Saudi woman with undifferentiated connective tissue disease based on joint pain, mainly ankle pain and fatigue, was positive for antinuclear antibody for 5 years and was on hydroxychloroquine, which was stopped. The patient presented with diffuse itchy skin eruptions all over her body, including the lower limb, upper limb, scalp, face, and buttocks, with erythema for 2 weeks. The patient had a history of fatigue and arthralgia. She presented with no history of skin rash on her palms or soles, oral ulcers, genital ulcers, hair loss, chest pain, shortness of breath, hemoptysis, constitutional symptoms, eye manifestations, abdominal pain, vomiting, hematuria, dysuria, previous thrombosis, or any neurological symptoms such as headache, numbness, or weakness. She reported no family history of autoimmune rheumatic disease. On examination, the patient appeared unwell, but she was conscious and oriented, and her vital signs were normal. Cutaneous examination revealed numerous papules and nodules with central keratotic plugs on her lower limbs, upper limbs, and face (Fig. 1). The systemic examination results were unremarkable. Laboratory investigations, including complete blood count, liver function tests, kidney function tests, and inflammatory markers, were normal. Furthermore, she had a positive antinuclear antibody 1:160 using enzyme-linked immunosorbent assay and anti-smooth muscle antibody 1:80 (<1:40) with negative anti-double stranded DNA antibodies and anti-phospholipid antibodies, a negative extractable nuclear antigen profile, negative antineutrophil cytoplasmic antibodies, anti-cyclic citrullinated peptide, and rheumatoid factor. The patient had normal complement levels and no proteinuria. Skin biopsy revealed reactive perforating collagen (Fig. 2). The patient was admitted and treated with systemic antihistamines (diphenhydramine 50 mg orally, twice daily), topical steroid cream (betamethasone dipropionate cream), and oral isotretinoin 20 mg daily. The patient was advised to undergo phototherapy. Following isotretinoin therapy, the patient’s skin rash cleared within 2 months, and there were no new skin lesions or systemic features. A year later, the patient developed Raynaud’s phenomenon, oral and nasal ulcers, malar rash, fatigue, and lupus rash around her eyes. SLE was diagnosed clinically, and the patient started taking oral hydroxychloroquine 200 mg twice daily. The patient is still undergoing routine follow-up visits to the rheumatology clinic.

## 4. Discussion and conclusion

RPC is characterized by the development of plaques and nodules with core keratins, which are typically found in the lower extremities and cause severe itching.^[2–4]^ It has frequently been challenging to identify the effective pathophysiology of the disease because its etiology is unknown. However, most scientists have suggested that the disease is caused by the excessive production of keratin and cellular components,^[6]^ and skin keratinization occurs in the basilar stratum, which is typically located below the proliferative layer of the epidermis.^[7]^ Another theory proposed by researchers is that the inflammatory reaction may be caused by changes in the connective tissues in the dermis, similar to the reactive response seen in other skin diseases as perforating collagenases.^[7]^ RPC is an inflammatory skin disease believed to spread via the excretion of keratin and other cellular components of the skin. In contrast, connective tissue diseases, such as SLE, are heterogeneous when the immune system falsely reacts in the body, which can cause different skin and vasculitis lesions that can be confused with other inflammatory skin diseases. Additionally, it is important to observe whether there is a clear association between RPC and autoimmune rheumatic diseases or whether it could be an initial presentation of SLE or other connective tissue diseases. Furthermore, the timing of the skin biopsy may be crucial for the diagnosis of RPC. Few cases of RPC have been reported in autoimmune rheumatic diseases such as vasculitis, SLE, and idiopathic myositis, although it might be challenging to determine whether it is an association or a complication^[8–11]^ (Table 1). Similar to our patient, Fujimoto et al reported that the soles, mucous membranes, and palms are immune to RPC.^[10]^ The treatment of the underlying and associated conditions is the most important measure. However, patients are recommended to try various drugs such as oral antihistamines, isotretinoin, emollients to alleviate itchiness, and phototherapy.

**Table 1:** Summary of reported perforating collagenosis cases and rheumatic diseases.

Author	Patient gender	Ethnicity	Age at presentation	Rheumatic disease	Journal	Year of publication
Muller, C.S et al	Female	Not mentioned	54 years	Leucocyto-clastic vasculiti	Dermato-Endocrinology	2009
Amano H et al	Female	Japanese	38 years	Dermatomyositis	The journal of Dermatology	2011
Ohashi, T et al	Female	Not mentioned	44 years	SLE	The journal of Dermatology	2016

SLE = systemic lupus erythematosus.

In conclusion, variable skin rashes can mimic SLE, other connective tissue diseases, and vasculitis. Therefore, RPC is a skin condition that requires high clinical suspension for diagnosis because the treatment approach differs.

## Acknowledgments

We sincerely appreciate the written consent of the patient.

## Author contributions

**Conceptualization:** Fahidah Alenzi.

**Data curation:** Fahidah Alenzi.

**Formal analysis:** Fahidah Alenzi.

**Writing – original draft:** Fahidah Alenzi.

**Writing – review & editing:** Fahidah Alenzi.

## References

[1] Faver IR Daoud MS Daniel Su WP. Acquired reactive perforating collagenosis: Report of six cases and review of the literature. J Am Acad Dermatol. 1994;30:575–80. 8157784 10.1016/s0190-9622(94)70065-6

[2] Yang H Liu W Chang JM. Acquired reactive perforating collagenosis. J Clin Dermatol. 2017;46:297–9.

[3] Eljazouly M Alj M Chahboun F. Acquired reactive perforating collagenosis: a case report. Cureus. 2021;13. 10.7759/cureus.13583 PMC8006497 33796426

[4] Nair P Jivani N Diwan N. Kyrle’s disease in a patient of diabetes mellitus and chronic renal failure on dialysis. J Fam Med Prim Care. 2015;4:284. 10.4103/2249-4863.154678 PMC4408719 25949985

[5] Thomas E Pawar B Thomas A. A prospective study of cutaneous abnormalities in patients with chronic kidney disease. Indian J Nephrol. 2012;22:116–20. 22787313 10.4103/0971-4065.97127 PMC3391808

[6] Viswanathan S Narurkar S Rajpal A. Rare presentation of Kyrle’s disease in siblings. Indian J Dermatol. 2008;53:85–7. 19881995 10.4103/0019-5154.41654 PMC2763716

[7] Lee SJ Jang JW Lee WC. Perforating disorder caused by salt-water application and its experimental induction. Int J Dermatol. 2005;44:210–4. 15807728 10.1111/j.1365-4632.2004.01988.x

[8] Ohashi T Yamamoto T. Acquired reactive perforating collagenosis associated with systemic lupus erythematosus. J Dermatol. 2016;43:1097–9. 27028318 10.1111/1346-8138.13357

[9] Müller CSL Tilgen W Rass K. Leucocytoclastic vasculitis associated with acquired reactive perforating collagenosis. Dermatoendocrinol. 2009;1:229–31. 20592796 10.4161/derm.1.4.9555 PMC2835880

[10] Fujimoto N Akagi A Tajima S. Expression of the 67-kDa elastin receptor in perforating skin disorders. Br J Dermatol. 2002;146:74–9. 11841369 10.1046/j.1365-2133.2002.04550.x

[11] Amano H Nagai Y Kishi C Ishikawa O. Acquired reactive perforating collagenosis in dermatomyositis. J Dermatol. 2011;38:1199–201. 21545494 10.1111/j.1346-8138.2011.01210.x

